# Effect of Gum Arabic on Oxidative Stress and Inflammation in Adenine–Induced Chronic Renal Failure in Rats

**DOI:** 10.1371/journal.pone.0055242

**Published:** 2013-02-01

**Authors:** Badreldin H. Ali, Isehaq Al-Husseni, Sumyia Beegam, Ahmed Al-Shukaili, Abderrahim Nemmar, Simone Schierling, Nina Queisser, Nicole Schupp

**Affiliations:** 1 Department of Pharmacology and Clinical Pharmacy, College of Medicine and Health Sciences, Sultan Qaboos University, Al-Khod, Muscat, Sultanate of Oman; 2 Department of Physiology, College of Medicine and Health Sciences, Sultan Qaboos University, Al-Khod, Muscat, Sultanate of Oman; 3 Department of Microbiology and Immunology, College of Medicine and Health Sciences, Sultan Qaboos University, Al-Khod, Muscat, Sultanate of Oman; 4 Department of Physiology, Faculty of Medicine, UAE University, Al Ain, United Arab Emirates; 5 Institute of Pharmacology and Toxicology, University of Würzburg, Würzburg, Germany; University of Tokushima, Japan

## Abstract

Inflammation and oxidative stress are known to be involved in the pathogenesis of chronic kidney disease in humans, and in chronic renal failure (CRF) in rats. The aim of this work was to study the role of inflammation and oxidative stress in adenine-induced CRF and the effect thereon of the purported nephroprotective agent gum arabic (GA). Rats were divided into four groups and treated for 4 weeks as follows: control, adenine in feed (0.75%, w/w), GA in drinking water (15%, w/v) and adenine+GA, as before. Urine, blood and kidneys were collected from the rats at the end of the treatment for analysis of conventional renal function tests (plasma creatinine and urea concentration). In addition, the concentrations of the pro-inflammatory cytokine TNF-α and the oxidative stress markers glutathione and superoxide dismutase, renal apoptosis, superoxide formation and DNA double strand break frequency, detected by immunohistochemistry for γ-H2AX, were measured. Adenine significantly increased the concentrations of urea and creatinine in plasma, significantly decreased the creatinine clearance and induced significant increases in the concentration of the measured inflammatory mediators. Further, it caused oxidative stress and DNA damage. Treatment with GA significantly ameliorated these actions. The mechanism of the reported salutary effect of GA in adenine-induced CRF is associated with mitigation of the adenine-induced inflammation and generation of free radicals.

## Introduction

Chronic kidney disease (CKD) is a serious global health problem, and is now considered a key determinant of the poor health outcomes of major noncommunicable diseases [1]. Several factors influence the onset and progression of this CKD, such as obesity, hypertension and diabetes mellitus. Beyond these factors, there is evidence of a pathophysiological role for inflammation and oxidative stress in CKD and its complications [2]. These two events are prominent features of CKD and its complications in humans [3], [4], [5], [6], [7]. Increased oxygen radical formation was found in CKD, in the presence of a reduced antioxidant defense [8]. Patients suffer from chronic microinflammation and infections [9]. Furthermore, markers of oxidative stress and inflammation are increased, like lipid peroxidation and glutathione content, or C-reactive protein (CRP) and IL-6 [10], [11], [12]. Oxidative stress and inflammation are also major mediators of the disease, exerting similar effects in the surgically-induced chronic renal failure (CRF) model in rats [13], [14]. Patients with CKD have high plasma concentrations of inflammatory mediators (such as CRP, tumor necrosis factor (TNF)-α and other cytokines) and several markers of oxidative stress [15], [16].

Gum arabic (GA, E-Number 414) is an edible, dried gummy exudate from the stems and branches of *Acacia senegal* and *Acacia seyal*, that is rich in non-viscous soluble fiber. It is widely used in pharmaceutical and food industry as an emulsifier and stabilizer [17]. For centuries it has been used as an oral hygiene substance by many communities in the Middle East and North Africa [18]. Also its anti-inflammatory properties were taken advantage of in folk medicine, where it was used internally to treat inflammation of intestinal mucosa and externally to cover inflamed skin [19]. For some time now GA is used in Arab folk medicine in patients with CRF [17], [20]. Experimentally, GA treatment has been shown to ameliorate some biochemical, physiological and behavioral effects in rats with adenine-induced CRF [21], [22], [23] and to modulate immunity in mice [24]. Clinically, GA has been shown to be beneficial in patients with CRF [25], and later, this was confirmed in patients with CRF in the Sudan, where it was claimed to help decrease urea and creatinine plasma concentrations and reduced the need for dialysis from 3 to 2 times per week [26]. Subsequently, similar results have been obtained from uremic children in Iraq [27] and uremic adults in central Sudan [28]. The basis of this salutary effect of GA on renal function is probably an urea-lowering effect through utilizing the bowel as a “substitute kidney”, increasing urea nitrogen (N) excretion in stools, with a concomitant decrease in the total N excreted in urine [17], [29], [30]. Sorbents (such as resins) can augment hemodialysis systems by adsorbing/removing conventional uremic toxins such as urea and creatinine, and also other toxins [30]. It has also been shown that butyrate modifies the generation of the pro-fibrotic cytokine transforming growth factor-beta (TGF-β1) by renal epithelial cells, and that dietary supplementation with a naturally processed polysaccharide exudate from *Acacia senegal* can increase serum butyrate, which, at least *in vitro*, has beneficial effects on renal pro-fibrotic cytokine generation [31].

In the present work, we have extended our previous observations on the effects of GA treatment on rats with adenine–induced CRF [21], by investigating the anti-inflammatory and antioxidant mechanisms related to the protective effect of GA on adenine-induced CRF, using several novel parameters such as IL-10, as well as the generation of reactive oxygen species and DNA strand breaks. The results of our study will further explain the mechanisms of the beneficial effects of GA.

## Results

### Gum Arabic Ameliorates Adenine-induced Renal Failure

**Figure 1:**
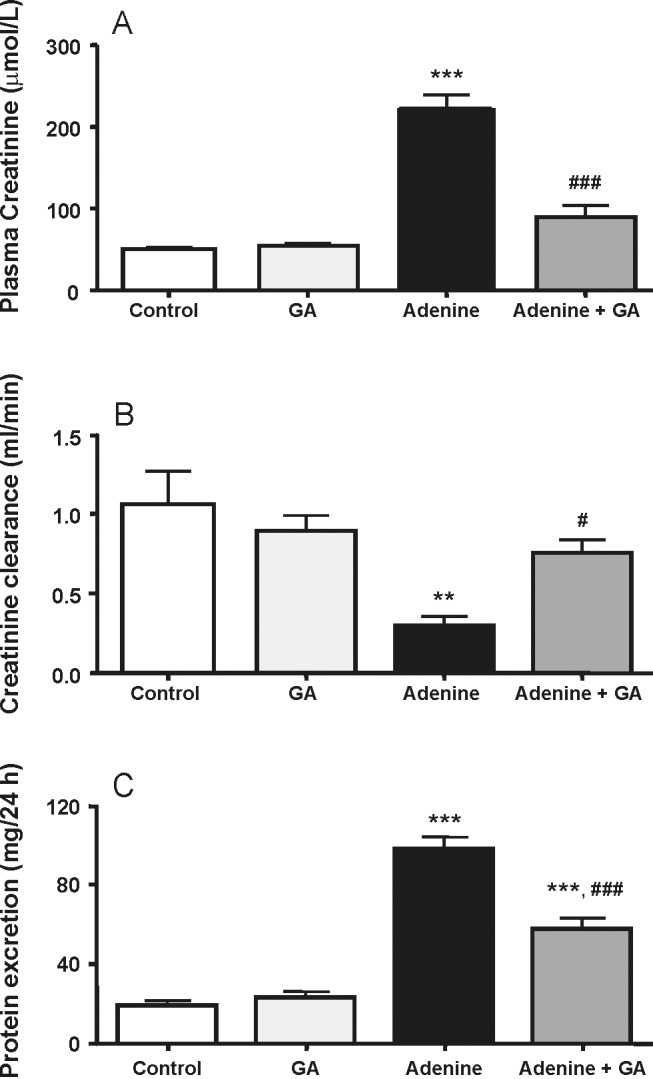
Plasma creatinine (A) and creatinine clearance (B), as well as proteinuria (C) in control rats, rats treated with gum arabic (15% w/v in drinking water) and rats treated with adenine (0.75% w/w) alone in feed, or with adenine and gum arabic given concomitantly at the same dose for 28 days. Each column and vertical bar represents the mean ± SEM (n = 6). ** p<0.01, *** p<0.001 vs. control, ^#^ p≤0.05, ^###^ p<0.001 vs. adenine treatment.

**Figure 2:**
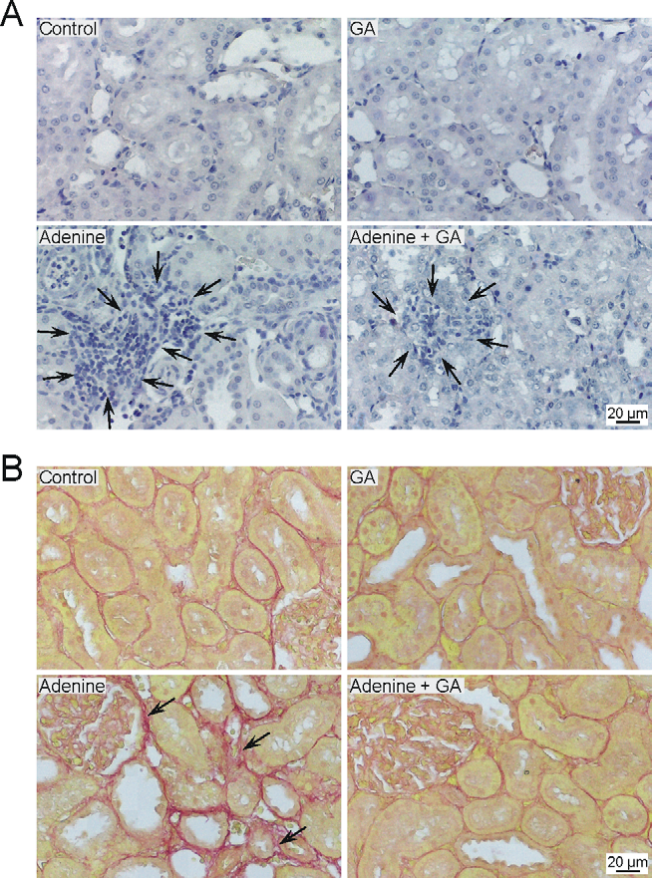
Occurrence of inflammation (A) in kidneys of control rats, rats treated with gum arabic (15% w/v in drinking water) and rats treated with adenine (0.75% w/w) alone in feed, or with adenine and gum arabic given concomitantly at the same dose for 28 days. Black arrows in the representative pictures of tissue stained with hemytoxylin point to leucocyte infiltration. Occurrence of fibrosis (B) in the kidneys of control rats, rats treated with gum arabic (15% w/v in drinking water) and rats treated with adenine (0.75% w/w) alone in feed, or with adenine and gum arabic given concomitantly at the same dose for 28 days. Shown are representative pictures of fibrosis in the kidney, with black arrows pointing to examples of collagen disposition, visualized by Sirius Red staining.

In the CRF model used in the present study, adenine is given mixed with the feed at a concentration of 0.75%, w/w, for 4 weeks. Orally administered adenine is metabolized to 2,8-dihydroxyadenine, which precipitates and forms tubular crystals that injure the renal tissue. We could confirm here the previously reported effects [21], [22] that adenine feeding (0.75% for 4 weeks) caused significant increases (P<0.001) in the concentrations of plasma creatinine and a significant decrease in the creatinine clearance (P<0.01) (Fig. 1). Treatment with GA significantly abated the adenine effect. As a further marker of kidney injury, proteinuria was analyzed (Fig. 1), showing a significant increase of excreted protein in adenine-treated rats, which was reduced significantly by GA, although not completely. Histopathological examination of the kidney revealed extensive signs of inflammation and fibrosis in kidneys of the adenine treated animals (Fig. 2 and Table 1), as well as glomerular damage (Table 1). GA significantly lowered this morphological damage (Table 1).

**Table 1:** Effect of treatment of rats with gum arabic (GA, 15% w/v in drinking water), with or without adenine in feed (0.75% w/w) for 28 days on histopathological parameters.

Group	GSI	MSI	Fibrosis	Inflammation
Control	0.46±0.10	0.35±0.07	0.19±0.02	0.06±0.01
GA	0.49±0.13^###^	0.41±0.10^###^	0.21±0.03^###^	0.05±0.01^###^
Adenine	1.85±0.40^***^	1.46±0.10^***^	2.12±0.10^***^	2.70±0.18^***^
Adenine+GA	1.00±0.10^**,###^	0.77±0.10^*,###^	0.39±0.03^###^	1.29±0.10^***,###^

The values represent the mean ± SEM (n  = 6). * p<0.05, ** p<0.01, *** p<0.001 vs. control, ^###^ p<0.001 vs. control vs. adenine treatment. GSI = glomerular sclerosis index, MSI = mesangiolysis index.

### Effect of Gum Arabic on CRP and TNF-α

**Figure 3:**
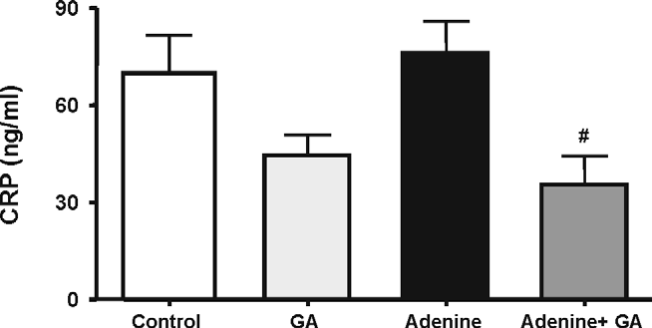
Plasma C-reactive protein concentration in control rats, rats treated with gum arabic (15% w/v in drinking water) and rats treated with adenine (0.75% w/w) alone in feed, or with adenine and gum arabic given concomitantly at the same dose for 28 days. Each column and vertical bar represents the mean ± SEM (n = 6). ^#^ p≤0.05 vs. adenine treatment.

**Figure 4:**
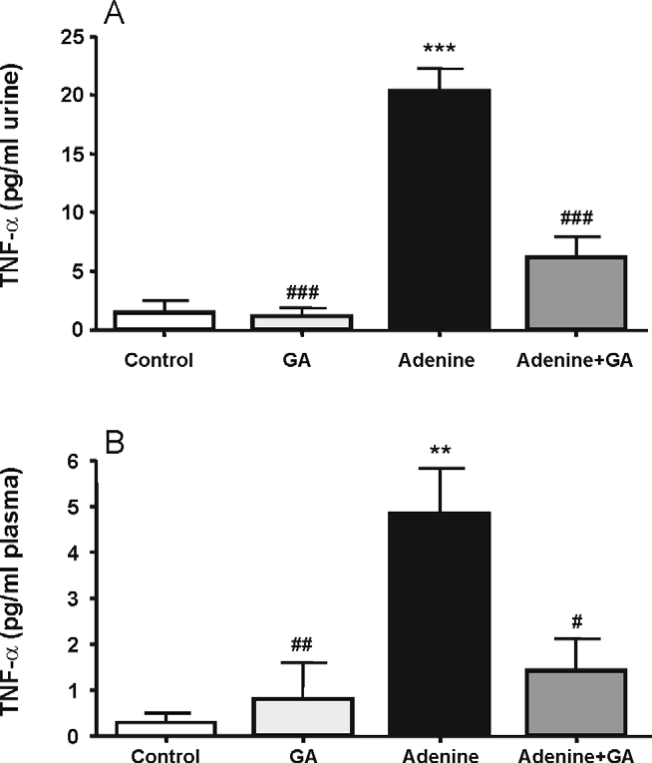
Tumor necrosis factor-α concentration in urine (A) and plasma (B) in control rats, rats treated with gum arabic (15% w/v in drinking water) and rats treated with adenine (0.75% w/w) alone in feed, or with adenine and gum arabic given concomitantly at the same dose for 28 days. Each column and vertical bar represents the mean ± SEM (n = 6). ** p<0.01, *** p<0.001 vs. control, ^#^ p<0.05, ^##^ p<0.01,^###^ p<0.001 vs. adenine treatment.

CRP was significantly (P<0.05) decreased to about 51% in rats treated with adenine plus GA, when compared with rats treated with adenine alone (Fig. 3). The TNF-α concentration in urine and plasma was significantly increased in the adenine-treated group, and this increase was markedly and significantly diminished in rats treated with both adenine and GA (Fig. 4).

### Effect of Gum Arabic on IL-10

**Figure 5:**
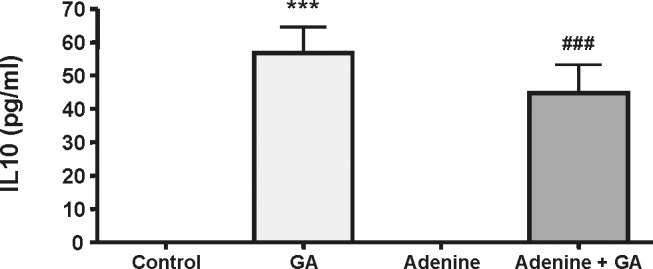
Interleukin 10 (IL-10) concentration in the plasma of control rats, rats treated with gum arabic (15% w/v in drinking water) and rats treated with adenine (0.75% w/w) alone in feed, or with adenine and gum arabic given concomitantly at the same dose for 28 days. Each column and vertical bar represents the mean ± SEM (n = 6). *** p<0.001 vs. control, ^###^ p<0.001 vs. adenine treatment.

The concentrations of the anti-inflammatory cytokine IL-10 were not detectable in rats treated with water (controls) and adenine (Fig. 5). However, the concentration of this cytokine was significantly increased in the GA-treated rats (P<0.001) compared to the control and the adenine-treated rats. In rats treated with GA and adenine, the concentration of IL-10 was not significantly different from those in rats treated with GA alone.

### Antioxidative Effects of Gum Arabic

**Figure 6:**
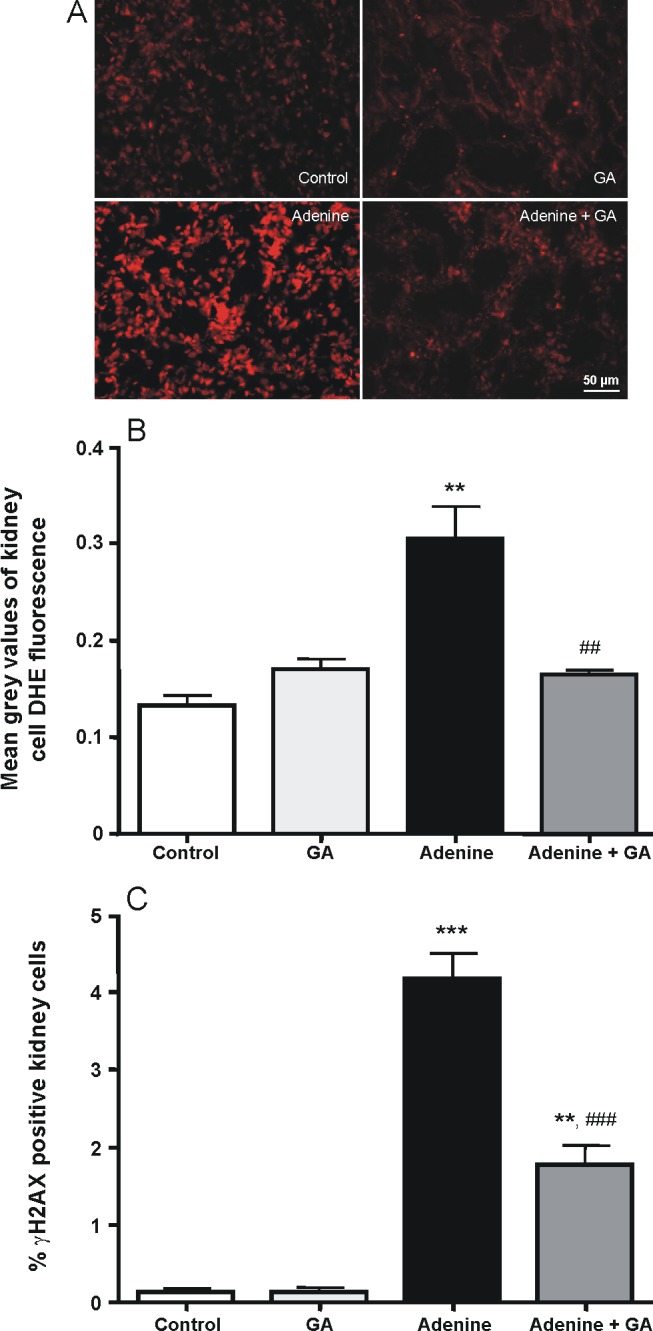
Representative pictures of superoxide formation visualized by using the dye dihydroethidium on kidney cryosections (A). Superoxide (B) and DNA double strand break formation (C) in control rats, rats treated with gum arabic (15% w/v in drinking water) and rats treated with adenine (0.75% w/w) alone in feed, or with adenine and gum arabic given concomitantly at the same dose for 28 days. Each column and vertical bar represents the mean ± SEM (n = 5). ** p<0.01, *** p<0.001 vs. control, ^##^ p<0.01, ^###^ p<0.001 vs. adenine treatment.

As shown in Fig. 6A and B, superoxide formation was significantly higher in the kidneys of adenine-treated rats compared to the kidneys of controls, GA or GA+adenine. GA decreased the superoxide production to control levels. DNA double strand breaks also were significantly increased in the kidney by adenine treatment (Fig. 6C). GA reduced this effect significantly, but was not able to restore control levels. Table 2 shows the concentrations of GSH and TAOA, as well as the SOD activity in the four groups. Adenine treatment significantly reduced the values of these analytes compared to controls and GA-treated rats (P<0.05). GA significantly ameliorated these actions in adenine-treated rats.

**Table 2:** Effect of treatment of rats with gum arabic (GA, 15% w/v in drinking water), with or without adenine in feed (0.75% w/w) for 28 days on indices of oxidative stress in renal cortex and plasma.

Group	GSH (µg/g)	SOD (U/g)	TAOA^a^(µg/l)
Control	7.21±0.45	1.36±0.13	0.61±0.09
GA	8.01±0.85^#^	1.86±0.19^#^	0.75±0.11^#^
Adenine	4.73±0.40^*^	0.89±0.10^*^	0.46±0.08^*^
Adenine+GA	6.01±0.46^#^	1.17±0.14^#^	0.54±0.07^#^

The values represent the mean ± SEM (n  = 6). * p<0.05 vs. control, ^#^ p<0.05 vs. adenine treatment.

Reduced glutathione (GSH) concentration and superoxide dismutase (SOD) activity were measured in renal homogenates, and total antioxidant activity (TAOA) was estimated in plasma.

a mM uric acid equivalents.

## Discussion

The worldwide incidence of CKD is increasing [32], but access to renal replacement therapy, either transplantaion or dialysis is limited in several regions of the world due to a lack of financial and clinical resources [33], [34]. Strategies to delay the onset of dialysis or to attenuate uremia often rely on dietary supplements. GA, traditionally used as an oral hygienic substance and to treat inflammation of intestinal mucosa and inflamed skin, was found to increase fecal N excretion and to lower serum urea nitrogen concentration in the 90′s [25], [29], and therefore is since then used in folk medicine to treat CKD [27]. Beside the increased clearance of nitrogen in CKD, GA has further beneficial effects on kidney function, which might be due to its anti-inflammatory and antioxidative effects as shown in this study.

Adenine-induced renal failure is the most often used chemically induced experimental animal model for CKD. Inflammation and oxidative stress in this experimental model, introduced three decades ago [35] have, as far as we are aware, not been studied in detail before. More is known about the involvement of oxidative stress and inflammation in the RKM model, as demonstrated by Kim et al. [36] for example. In the present study, as in our previous work, adenine treatment induced all the classical signs of renal impairment reported earlier [21], [22]. For brevity, in this work we reported the effects of adenine on plasma creatinine, creatinine clearance, and proteinuria. GA has been shown to act as an antioxidant, and to modulate inflammatory and/or immunological processes [17]. For example, the cytoprotective effects of GA against cisplatin-induced nephrotoxicity and cyclophosphamide-induced urinary bladder cytotoxicity in rats have been ascribed to a scavenging action against reactive oxygen metabolites [37], [38]. GA has also been reported to have a partial ameliorating action against experimental gentamicin-induced nephrotoxicity in rats [39].

In the present work, we tested in renal tissue, plasma and urine of rats, the effect of GA treatment (15% in the drinking water for 4 weeks) on several established inflammatory and oxidative stress markers in rats with adenine–induced CRF. It is known that samples of different GA products can be inherently variable, depending on their sources and location. Here, we have used an *Acacia senegal var. senegal* sample, which has been matured to yield a standardized and reproducible test material, with a known molecular weight [31].

As a sign of inflammation, tissue infiltration of white blood cells was observed at histopathological examination of kidneys of adenine-treated animals, which was significantly suppressed in animals treated with adenine together with GA. CRP is an acute phase reactant that is increased in inflammation and infection, and has long been used as a biomarker indicating these conditions [40]. It has been shown to be increased in plasma of RKM rats [41]. Our results show that co-administration of GA to adenine-treated rats resulted in a significant reduction in plasma CRP concentration, although GA treatment alone was not effective in altering its level. Just recently, Mahmoud et al [42] reported that rats fed with adenine for 8 weeks (longer than the usual 4 weeks), increased the concentration of serum C-reactive protein and a few antioxidant parameters, and that GA mitigated these action. CRP is known as a mediator stimulating the release of other pro-inflammatory cytokines such as IL-6 and TNF-α [43]. Treatment with adenine induced a marked rise in TNF-α, which is largely in concordance with the results of the other quantified cytokines.

IL-10 is known to act in different cell types where it suppresses inflammatory responses [44]. One of the most striking findings in this study was that treatment with GA alone induced a significant rise in plasma IL-10 concentration. Co-administration of GA and adenine slightly reduced the concentration of this anti-inflammatory cytokine. A direct evidence for an anti-inflammatory action of GA, like the induction of IL-10, has not, as far as we know, been reported. However, GA boosts immunity in mice [24], and induces an apparent anti-inflammatory action when used against gingival inflammation [45]. It has also recently been reported, that dietary supplementation with soluble fibers suppresses gut inflammation in IL-10-deficient mice [46].

Reactive oxygen species directly impair mitochondrial function, protein synthesis and structure, DNA synthesis and cellular repair mechanisms [47]. Oxidative stress is already found in early stages of renal disease and increases with declining kidney function [48]. In adenine-induced CRF, until now oxidative stress was demonstrated in the heart and in the vasculature [49], [50], so this is the first account of increased superoxide production in the kidneys. DNA damage in kidney disease was first detected in the DOCA/salt model, where DNA single and double strand breaks were found [51]. Therefore, the adenine-induced CRF model used here is only the second renal failure model in which DNA damage has been analyzed. In both models the source of the DNA damage seems to be increased oxidative stress. The antioxidative capacity of GA could prevent the formation of superoxide completely and the oxidative stress-induced DNA double strand breaks to a certain extent. DNA double strand breaks are serious lesions, initiating genomic instability, inducing cell death or even mutations [52]. A lowered amount of superoxide anions and a lowered incidence of double strand breaks could in part explain the positive effect of GA on the progression of kidney disease. This positive effect can possibly also be ascribed to the ability of GA to lower the blood pressure in the adenine-treated rats [23], as we and others showed an increase of ROS in animals with hypertension [51], [53], [54].

In conclusion, this work provides direct evidence of anti-inflammatory and antioxidative capacities of GA. GA was able to decrease high levels of several pro-inflammatory cytokines in plasma and kidney of rats suffering from adenine-induced CRF. Further, it could ameliorate a loss of antioxidant defense and decrease adenine-induced superoxide production and DNA double strand breaks, two damage parameters shown for the first time in this CRF model. These anti-inflammatory and antioxidative capacities of GA add to the explanation of its beneficial actions as a dietary supplementation in patients suffering from CKD.

## Materials and Methods

### Animals

Male Wistar rats (9–10 weeks old, weighing 249±10 g) were housed in a room at a temperature of 22±2°C, relative humidity of about 60%, with a 12 h light–dark cycle (lights on 6∶00), and free access to standard pellet chow diet containing 0.85% phosphorus, 1.12% calcium, 0.35% magnesium, 25.3% crude protein and 2.5 IU/g vitamin D3 (Oman Flour Mills, Muscat, Oman) and water. Procedures involving animals and their care were carried out in accordance with international laws and policies (EEC Council directives 86/609, OJL 358, 1 December, 12, 1987; NIH Guide for the Care and Use of Laboratory Animals, NIH Publications No. 85–23, 1985), and ethical clearance was obtained from the Small Animal Research Ethics Committee of Sultan Qaboos University.

### Experimental Design

After an acclimatization period of one week, rats (n = 24) were randomly divided into four equal groups and treated for four consecutive weeks. The first group continued to receive the same diet without treatment until the end of the study (control group). The second group was switched to a powder diet containing adenine (0.75%^w/w^ in feed). The third group was given normal food and GA (SUPERGUM™ EM 10) in drinking water at a concentration of 15% w/v. The fourth group was given adenine in the feed as in group two, plus GA in drinking water at a concentration of 15% w/v. The dose of adenine was chosen from the original method by Yokozawa et al. [55] and the dose of GA was chosen on the basis of our previous experiments with GA [21], [23]. It was slightly increased in this and other subsequent studies [56], in order to maximize the effect of GA.

During the treatment period, the rats were weighed weekly. For the collection of urine, they were placed individually in metabolic cages for 24 h, after the 28 days treatment period. On the morning after the metabolic sampling, the rats were anesthetized with an intraperitoneal injection of ketamine (75 mg/kg) and xylazine (5 mg/kg), and blood (about 3.5 mL) was collected from the anterior vena cava and placed into heparinized tubes. The blood and urine were centrifuged at 900 g at 4°C for 15 min. The plasma obtained, together with the urine specimens, was stored at −80°C to await analysis within 4 weeks after the end of the treatment. The two kidneys were excised, blotted on filter paper and weighed. The rest of the kidneys were kept frozen at −80°C for pending biochemical analysis within three days. The left kidney was homogenized in ice-cold Tris buffer (pH 7.4) to give a 10% w/v homogenate. The latter was centrifuged at 1500 g at 4°C for 15 min, and the supernatant obtained was used to measure glutathione (GSH), and superoxide dismutase (SOD) activity.

### Biochemical Methods

The concentrations of creatinine in plasma and urine were estimated spectrophotometrically using commercial kits (BioMerieux, Marcy-l’Etoile, France). Creatinine clearance (CCr) was calculated as reported by Duarte et al. [57]. Proteinuria was measured with a kit from HUMAN GmbH (Wiesbaden, Germany). Total antioxidant activity (TAOA) was measured in serum using a kit from Oxford Biomedical Research (Oxford, MI, USA). In renal cortex homogenates, glutathione (GSH) concentration was measured with a spectrophotometric method [58], and superoxide dismutase (SOD) activity with a kit from Randox, Antrim, UK. C-reactive protein (CRP) was measured using an ELISA kit from GenWay Biotech, Inc. (San Diego, CA, USA), respectively. Tumor necrosis factor alpha (TNF-α) and interleukin-10 (IL-10) ELISA kits were from R&D Systems Europe Ltd (Abingdon, UK).

### Histopathology

For histopathological investigation of the kidney 2 µm sections were cut and stained with hematoxylin, periodic acid-Schiff stain (PAS) or Sirius Red stain. In the kidneys the glomerular sclerosis index (GSI) and the mesangiolysis index (MSI) were determined as described in [59]. Fibrosis was seperately evaluated on Sirius Red stained slides and inflammation on hematoxylin-stained slides within 40 (fibrosis) or 80–100 (inflammation) visual fields using a semiquantitative scoring ranging from 0 to 4 (grade 0∶0% fibrosis, grade 1: <25% fibrosis, grade 2 25–50% fibrosis, grade 3∶50–75% fibrosis, grade 4: >75% fibrosis).

### Immunohistochemistry for γ-H2AX (Measurement of DNA Double Strand Breaks)

Frozen kidney sections (5 µm) were transferred from −80°C to be stored for 20 min in −20°C. The sections were fixed in 4% formaldehyde for 15 min at room temperature and afterwards for 5 min in methanol at −20°C. Hydrogen peroxide (3% in methanol) was applied for 10 min, followed by incubation for 1 h at room temperature in 10% normal donkey serum (Chemicon, Amersfoort, The Netherlands). Phospho-Histone H2A.X (Ser139)(20E3) Rabbit monoclonal Ab (Cell Signaling, Danvers, USA; 1∶200) was applied and incubated overnight at 4°C. Sections were then rinsed in PBS and incubated with rhodamine-conjugated donkey anti-rabbit secondary antibody (Santa Cruz, Santa Cruz, USA; 1∶100) for 30 min at room temperature. After washing in PBS/Tween [0.2% v/v] for 5 min, the sections were counterstained with the DNA stain bisbenzimide (AppliChem, Darmstadt, Germany; 10 µg/ml) for 3 min. Sections were washed with PBS and mounted with Confocal Matrix (Micro Tech Lab, Graz, Austria). Immunofluorescent images were captured using an Eclipse55i microscope (Nikon GmbH, Düsseldorf, Germany) and a Fluoro Pro MP 5000 Camera (Intas Science Imaging Instruments GmbH, Göttingen, Germany) at a 200-fold magnification. Images excited at 465–495 nm for positive γ-H2AX foci (red fluorescence) were merged with those excited at 330–380 nm for bisbenzimide (blue fluorescence). For quantification, 8 non-overlapping microscopic fields of renal cortex were analyzed by the cell image analysis software CellProfiler (Broad Institute, Cambridge, USA).

### Measurement of Superoxide Formation

Superoxide production on 5 µm cryosections (Leica CM 3050 S, Leica Microsystems, Wetzlar, Germany) was detected after staining the sections for 20 minutes with 10 µM dihydroethidium (Merck, Darmstadt, Germany) at room temperature in the dark. Pictures were taken with an Eclipse 55i microscope (Nikon GmbH, Düsseldorf, Germany) at a 200-fold magnification. Quantification was done with CellProfiler (Broad Institute, Cambridge, USA) by measuring gray values in 8–12 non-overlapping microscopic fields.

### Drugs and Chemicals

Acacia gum used: SUPERGUM™ EM 10, Lot 101008, 1.1.11 (Sanwa_Cho, Toyonaka, Osaka, Japan). Adenine was obtained from Sigma (St. Louis, MO, USA). Aqueous solutions of both compounds were prepared freshly every day. The chemical properties of GA have been fully reviewed before [17]. All used chemicals were of analytical reagent grade.

SUPERGUM™ EM 10 was characterized by size fractionation followed by multiple angle laser light scattering (GPC-MALLS) to give its molecular profile. The average molecular weight was 3.43×10^6^, and the content of the arabinogalactan protein (AGP) was 26.4%.

### Statistics

Statistical analysis was carried out using GraphPad Prism 4.0 (GraphPad Software, San Diego, CA, USA) or SPSS Statistics 19 (IBM, Ehningen, Germany). Each group consisted of 6 animals. All data are expressed as means ± S.E.M. Group means were compared with an analysis of variance (ANOVA) followed by Tukey’s multiple comparison test. Values of p<0.05 were regarded as significant.
